# The Virtual-Body Project Reduces Eating Disorder Symptoms Among Young Adult Brazilian Women: A Pilot Study

**DOI:** 10.3390/healthcare13111329

**Published:** 2025-06-03

**Authors:** Karin Louise Lenz Dunker, Ana Carolina Soares Amaral, Pedro Henrique Berbert de Carvalho

**Affiliations:** 1 Eating Disorders Program, Institute of Psychiatry (AMBULIM), University of São Paulo, São Paulo 05403-010, SP, Brazil; kdunker00@gmail.com; 2 Federal Institute of Education, Science and Technology of Southeast of Minas Gerais, Barbacena 36205-018, MG, Brazil; ana.amaral@ifsudestemg.edu.br; 3 Body Image and Eating Disorders Research Group (NICTA), Federal University of Juiz de Fora, Governador Valadares 35010-180, MG, Brazil

**Keywords:** eating disorders, body dissatisfaction, negative affect, sociocultural influences, self-esteem, body appreciation, prevention, intervention

## Abstract

**Background/Objectives:** Dissonance-based (DB) eating disorder (ED) prevention programs have been showing their efficacy in reducing ED symptoms among adolescents, young adults, and older people. Several meta-analyses showed that the Body Project is the most effective ED prevention program for at-risk women; however, the program presents high costs when delivered in-person and recruitment on a large scale is limited, suggesting the evaluation of its efficacy when delivered virtually. Thus, we investigated the efficacy of the v-Body Project (i.e., a virtual DB ED prevention program) among young adult Brazilian women. **Methods**: A pilot study delivered the v-Body Project to 85 Brazilian women (M_age_ = 22.55, SD = 2.07, age range = 18–25). Measures of ED symptoms, body dissatisfaction, the thin ideal internalization, negative affect, self-esteem, and body appreciation were applied at baseline, post-intervention, and at 1-month and 6-months follow-up. **Results**: Results demonstrated improvements in all outcomes at post-intervention. Large effect sizes were found for ED symptoms, body dissatisfaction, thin–ideal internalization, negative affect, and body appreciation (Cohen’s d = 0.74–1.31) and were maintained up to 6-months. A small effect size was identified for self-esteem (Cohen’s d = 0.40), while the efficacy was maintained up to 6-months. **Conclusions**: Results support the efficacy of the v-Body Project up to 6-months, providing a tool with lower costs for participants and the advantage of large-scale application for ED prevention programs. Strategies are needed to implement this protocol within the Brazilian public health system, including the training of facilitators and the broader dissemination of the intervention.

## 1. Introduction

People of all ages, socioeconomic backgrounds, gender identities, and cultures are susceptible to eating disorders (EDs) [1]. EDs include threshold and subthreshold anorexia nervosa, bulimia nervosa, and binge eating disorder, which are linked to increased emotional suffering, functional impairment, and increased mortality [2,3,4]. A meta-analysis found that 14.9% (95% CI = 12.8–17.2%) of Brazilians are at risk of EDs [5], showing that disordered eating behaviors, a risk factor for EDs, are common among Brazilian men and women. Among Brazilian women the prevalence of binge eating, purging, and food restriction were 4.4%, 2.5%, 9.1%, respectively [6], and these values demonstrate a temporal trend of increasing prevalence [7].

Notably the prevalence of EDs is raising worldwide [8]. Between the years of 2000–2018 the prevalence of EDs rose from 3.5% to 7.8%, drawing attention to a significant issue facing public health and medical professionals [8].

As per Swanson et al. [9], only 20% of people with EDs receive treatment. Furthermore, less than half of patients are able to achieve a sustained remission from their disordered eating behaviors [10,11]. Moreover, EDs have a large medical cost [12]. In order to lessen ED symptoms and the likelihood that EDs will recur in the future, it is imperative to widely adopt ED preventive programs.

The Body Project, a dissonance-based (DB) ED prevention program, is delivered in-person, and consists of a one-hour group session during four consecutive weeks [13]. The cognitive dissonance theory proposes that individuals experience psychological discomfort (dissonance) when they hold two or more contradictory beliefs, values, or attitudes, especially when their behavior conflicts with their beliefs. This discomfort motivates individuals to reduce the inconsistency, either by changing their beliefs, modifying their behavior, or rationalizing the discrepancy. The Body Project uses cognitive dissonance principles by having participants actively critique the thin ideal promoted by media and culture through verbal, written, and behavioral exercises [13]. By publicly arguing against the thin ideal, participants experience dissonance if they continue to internalize those standards themselves. To resolve this discomfort, they often shift their attitudes to align more with body acceptance and reject unrealistic beauty standards, which in turn lower risk factors for EDs [13,14,15].

The Body Project is also supported by the Dual-Pathway Model. According to this model, the internalization of the thin ideal and the pressure to be thin contribute to increased body dissatisfaction and, consequently, to the adoption of risk behaviors such as dieting and negative affect, which may lead to the onset of EDs. The authors found that changes in the thin ideal internalization fully mediated the program’s effects on ED symptoms [13].

Research has shown that the Body Project is very effective in lowering ED symptoms and risk factors (e.g., the thin ideal internalization and body dissatisfaction), as well as preventing future ED onset among young women [14]. When compared to control individuals, participants in the Body Project have demonstrated a 60% reduction in the likelihood of developing EDs in the future [15], and when compared to alternative therapies, this reduction is between 54% and 58% [13].

Among adolescent Brazilian girls [16], participants of the Body Project demonstrated significant decreases in depressive symptoms, negative affect, body dissatisfaction, and the sociocultural influence of the media, as well as significantly greater increases in body appreciation when compared with the assessment-only control group. Similar results have been obtained with young adult Brazilian women [17,18]. For example, Hudson et al. [17] found significantly higher increases in body appreciation and significantly lower levels of body dissatisfaction, sociocultural influence, disordered eating, ED symptoms, depressive symptoms, and negative affect in participants of the Body Project compared to an assessment-only control group [17]. The effects lasted up to 6 months [17]. Interestingly, DB ED prevention programs have shown significant effects in reducing ED symptoms not only in girls and young women [19,20,21]. Notwithstanding its large effects, the efficacy and effectiveness of the Body Project and other DB ED prevention programs (e.g., Body Project: More than Muscle [19,20] and the PRIDE Body Project [21]), it is necessary to point out limitations regarding its implementation. First, it is only offered in-person to groups of students in high school or college by qualified facilitators (e.g., peer educators or college counselors). Therefore, there is a need for participants to commute to participate in the groups, which is costly [22]. Second, based on past experiences, having about eight participants is crucial to guarantee that everyone can take part fully and still have enough one-on-one time. Then, recruitment on a large scale is limited [22]. As a result, Stice et al. [23] developed an unmoderated, individual internet version of the Body Project (i.e., the e-Body Project). However, in contrast to the group face-to-face Body Project, the effects were weaker and demonstrated less persistence over follow-up [23].

In order to preserve the group dynamics between members and facilitators and enhance scalability, Ghaderi et al. [22] assessed the outcomes of virtually implemented Body Project groups (i.e., v-Body Project). Psychology students received training on how to conduct the intervention in small groups of four to six participants (i.e., body-dissatisfied Swiss adolescents girls aged 15 to 20 years). Anybody who has access to the internet can participate in virtual group meetings, regardless of where they live. Ghaderi’s study [22] showed that in comparison to the waitlist participants at post-intervention and 6-months follow-up, as well as to the expressive writing participants at post-intervention and 6-, 12-, 18-, or 24-month follow-up, the v-Body Project participants generally demonstrated a significantly greater reduction in ED symptoms, body dissatisfaction, and the thin ideal internalization. The authors conclude that the v-Body Project could be a useful tool for evaluating scalable ED prevention in the future.

However, to date, few studies have evaluated the efficacy of the v-Body Project in different cultural contexts [24,25,26]. Thus, conducting a pilot study we aimed to assess the efficacy of the v-Body Project [22] among body-dissatisfied Brazilian women. Based on previous intervention studies with young adult Brazilian women [17,18], we hypothesized that the v-Body Project would demonstrate significant reduction in ED symptoms, body dissatisfaction, the thin ideal internalization, and negative affect (Hypothesis 1). We also hypothesized that the v-Body Project would demonstrate significant increases in self-esteem and body appreciation (Hypothesis 2).

It is worth noticing that in Brazil, since 2005, a nationwide survey known as *TIC Domicílios* has been conducted to map access to information and communication technologies in permanent households and their use by individuals aged 10 years and older. The 2024 survey results indicate that 85% of urban households in Brazil have internet access, while 74% of rural households are also connected. These data indicate that internet coverage in the country is adequate, suggesting the feasibility of implementing ED prevention programs delivered virtually, such as the v-Body Project.

## 2. Materials and Methods

### 2.1. Study Design and Participants

This was a non-experimental single group repeated measurement field study. Participants were assigned to the Body Project delivered through virtual groups (the v-Body Project). As this was a pilot study no waitlist control condition was included.

Inclusion criteria were to be female with body image concerns (a subjective sense of body dissatisfaction [i.e., higher than 5] in a self-rated scale ranging from 1 to 10) aged 18–25 years. Exclusion criteria were a current or past diagnosis of an ED, and/or had a score in the SCOFF (Sick, Control, One Stone, Fat, Food Questionnaire) [27] equal or higher than 3, and/or an EAT-26 (Eating Attitudes Test-26) score greater than or equal to 21 [28]. Women that filled these exclusion criteria were considered as at high risk for EDs and were informed about how to receive professional help.

Four hundred and thirty individuals expressed interest in the study. Of those, 274 were excluded based on eligibility criteria, for being in the at risk group or being over 25 years old. Although a total of 156 young adult Brazilian women were eligible to participate, only 85 connected at the first session and were eligible for analysis in the study.

### 2.2. Procedures

The original script of the v-Body Project [22] was translated by the authors and adapted to Brazilian Portuguese. Only minimal changes were made to improve cultural fit, and were based on changes in wording, expressions, and examples used in the role-play activities. Then seven health professionals (all were young adult Brazilians and self-identified as woman) were trained by the researchers involved in previous studies regarding the efficacy of the face-to-face version of the intervention (A.C.S.A and P.H.B.d.C.). All professionals had some prior knowledge of EDs. In addition to adherence to the intervention protocol, skills such as facilitating group discussions, responding to unexpected situations, and managing the timing of activities were also considered. Furthermore, they were required to have additional availability to monitor the receipt of participants’ submitted assignments and to arrange make-up sessions for any missed meetings.

Initially, the professionals participated in the intervention as participants themselves, in order to become familiar with the intervention model. This initial participation was not included in the training hours. Subsequently, they took part in the training, which consisted of 10 h of virtual meetings. In the first session, the theoretical foundations of the program were presented in detail. During the following four sessions, participants were trained to deliver each of the activities outlined in the protocol. The study was approved by the Internal Review Board of the Federal Institute of Education, Science and Technology of the Southeast of Minas Gerais and followed the ethical standards of the Declaration of Helsinki.

The program, named “Body Project Brazil” (BPB), had a partnership with the non-governmental organization the Brazilian Association of Eating Disorders (ASTRALBR). They promoted a national social media campaign (Instagram and Facebook) with the call: “Don’t you like what you see in the mirror? If you are a woman aged 18 to 25 and care a lot about your appearance and body, then come join the program Body Project Brazil. Come talk to us about the impacts of the media on our body satisfaction”. Participants signed up to groups by filling out a Google Form that included informed consent for participation and a questionnaire. Participants completed questionnaires at baseline, post-intervention, and at 1- and -6 months follow-ups.

The program was delivered from July 2022 to November 2023. During this time, six recruitment campaigns were publicized on social media. Fourteen groups were conducted with 6–8 participants at the first session. Each group was led by two facilitators. As soon as the research coordinator formed a group, trained professionals received contact information for the participants to schedule a first meeting.

All sessions were recorded for both supervision and fidelity ratings. Evaluating recorded sessions directly measured moderators’ fidelity to the protocol’s structure, content, and delivery. This process helped identify any deviations from the expected implementation. Feedback from the Body Project’s authors provided crucial insights into essential session elements and key messages. The combined approach of session evaluation and author feedback created a robust method for monitoring moderator adherence. This rigorous process was key to ensuring the virtual program’s high fidelity to the original protocol. Ultimately, strong moderator adherence, validated through these methods, enhanced the virtual program’s internal validity and overall effectiveness.

### 2.3. Intervention

The intervention comprised four sessions of one hour each, conducted over four consecutive weeks, as detailed by Stice et al. [15]. The initial session (Session 1) commenced with a group discussion centered on the thin body ideal and its related detriments. Subsequently, participants were tasked with completing homework assignments, which included composing a letter to a younger women outlining the adverse effects of pursuing the thin body ideal, and engaging in a mirror exercise to document positive self-attributes. Session 2 involved a review of the assigned homework and the lists of positive body qualities, the oral presentation of the letters, and participation in role-playing scenarios aimed at discouraging the facilitator from pursuing the thin body ideal. Participants were also given supplementary homework, such as generating a top-10 list of strategies to challenge prevailing thin or beauty ideals and writing a letter to an individual who had exerted pressure on them to conform to the thin body ideal, articulating the impact and their current viewpoint. Session 3 concentrated on the examination of homework assignments, the enactment of role plays to deflect comments related to the thin body ideal, the discussion of personal body image concerns, and engagement in further homework, encompassing body activism activities and behaviors designed to address body image challenges. The final session, Session 4, entailed a discussion of homework exercises, the development of strategies to resist future pressures to be thin through role playing, reflection on the advantages of the group experience, a commitment to self-affirmation exercises, and the composition of a letter to a younger female advising against body image concerns and encouraging participation in body activism. Each session commenced with a concise statement underscoring the voluntary nature of program participation.

### 2.4. Measures

#### 2.4.1. Demographics

Participants were asked to provide their demographic data consisting of age (open-ended), subjective sense of body dissatisfaction (self-rated scale ranging from 1 to 10), Brazilian region (North, North East, South, Southeast, or Midwest), current or past diagnosis of EDs (yes or no), self-reported anxiety symptoms (yes, no, or do not know/not reported), and self-reported depression symptoms (yes, no, or do not know/not reported).

#### 2.4.2. Eating Attitudes Test-26 (EAT-26)

For the evaluation of ED symptoms, the Eating Attitudes Test (EAT-26) [29] was employed. Across 26 statements (for example, “Vomit after eating” and “Engage in dieting behavior”), participants indicated the frequency of disordered eating behaviors and attitudes on a 4-point Likert-type scale (0 = never, rarely, sometimes; 1 = often; 2 = usually; 3 = always). The total scores ranged from 0 to 78, with scores above 21 indicating an elevated ED risk (i.e., a greater frequency of disordered eating correlates with higher scores). Among Brazilian women [28], the EAT-26 has demonstrated adequate validity and reliability. In the present study, the internal consistency (McDonald’s omega [ω]) for the EAT-26 ranged from 0.85 to 0.90 across the time points.

#### 2.4.3. Body Shape Questionnaire: Brief Version (BSQ-8)

For the purpose of evaluating body dissatisfaction, an abbreviated eight-item version of the Body Shape Questionnaire (BSQ-8), which has previously shown satisfactory psychometric properties [30], was used in this investigation. Participants were asked to rate each of the eight items on a 6-point Likert-type scale, with response options ranging from “never” to “always”, allowing for a nuanced assessment of their feelings. Consequently, the total score obtainable on the BSQ-8 ranges from 8 to 48, where elevated scores are indicative of a greater degree of body dissatisfaction experienced by the individual. The Brazilian adaptation of the BSQ-8 has been shown to possess good convergent validity and internal consistency, specifically among young adult women residing in Brazil [31]. Within the context of the current study, the internal consistency of the BSQ-8, as measured by appropriate statistical indices, ranged from 0.85 to 0.90 across the various time points at which data were collected, suggesting a robust reliability of the instrument throughout the study.

#### 2.4.4. Sociocultural Attitudes Towards Appearance Questionnaire-3 (SATAQ-3)

The Sociocultural Attitudes Towards Appearance Questionnaire-3 (SATAQ-3) [32] was used to assess sociocultural influences on body image. It includes four subscales and each item is rated on a 5-point Likert scale (1 = definitely disagree to 5 = definitely agree): general internalization, the athletic ideal internalization, pressures, and media as an appearance information source. The total score, calculated from all 30 items, ranges from 30 to 150, with higher scores indicating stronger sociocultural influence. Adequate validity and reliability for the SATAQ-3 have been shown in young Brazilian women [33]. In this study, McDonald’s ω for the SATAQ-3 ranged from 0.86 to 0.91 across time points.

#### 2.4.5. The Positive and Negative Affect Schedule–Revised (PANAS)

The Positive and Negative Affect Schedule (PANAS) [34] assessed positive and negative affect. In the current study, the negative affect subscale was applied, consistent with prior Body Project trials. Participants rated the intensity of emotions experienced in the past week on a 5-point Likert scale (1 = not at all/very slightly to 5 = extremely). The Portuguese version of the PANAS was used, following translation and back-translation procedures [35]. Total scores range from 10 to 50, with higher scores indicating greater negative affect. In this study, the internal consistency for negative affect ranged from 0.89 to 0.93 across time points, demonstrating good reliability.

#### 2.4.6. Rosemberg Self-Esteem Scale (RSES)

Self-esteem was assessed using the 10-item Rosenberg Self-Esteem Scale (RSES) [36], a validated self-report questionnaire with responses on a Likert-type scale (“completely agree” to “completely disagree”). Total scores range from 0 to 30, with higher scores indicating greater self-esteem. Reverse-scored items are included to reduce bias. The Brazilian version of the RSES, measuring positive and negative self-feelings, has shown good validity and internal consistency [37]. In this study, the RSES demonstrated good internal consistency across all time points (ω = 0.89–0.92).

#### 2.4.7. Body Appreciation Scale (BAS)

The 13-item Body Appreciation Scale (BAS; [38]) measures positive body perceptions, including attributes such as “feeling that my body has at least some good qualities”. Participants responded to each item on a 5-point Likert-type scale (1 = never to 5 = always), resulting in a total score ranging from 13 to 65. Higher scores indicate greater body appreciation. Swami et al. [39] conducted a psychometric evaluation of the BAS among Brazilian adult men and women. In the present study, the internal consistency of the BAS ranged from 0.95 to 0.96 across time points.

### 2.5. Data Analysis

The mean and standard deviation of numerical data, as well as the absolute and relative frequencies of categorical variables, were calculated. The expectation maximization method was used to handle missing data (Little’s test; *p* > 0.05) [40], and analyses included all individuals who completed any assessments [41]. The JASP software v. 0.19.1 [42] was used for all analyses, and a significance level of 5% (*p* < 0.05) was chosen. Using the ANOVA module (i.e., ANOVA for repeated measures), intent-to-treat analyses were performed to look at the long-term impact of the intervention on primary and secondary outcomes. Mauchly’s sphericity test was applied, and the residuals’ normal distribution assumptions were assessed. Greenhouse–Geisser correction was used (all Mauchly’s W were < 0.75) [43].

ANOVA for repeated measures effect size was reported by partial eta-squared (partial-η^2^). Values of 0.01–0.06, 0.06–0.14, and >0.14 were considered small, medium, and large, respectively [44]. Cohen’s d effect sizes were presented together with a time-series analysis of the differences between the calculated marginal means. Values were categorized as follows by Cohen [44]: 0.20–0.50 (small), 0.50–0.80 (medium), 0.80–1.00 (large), and very large (above 1.00).

## 3. Results

Table 1 presents demographic information of the Brazilian young women who took part in the v-Body Project (i.e., BPB).

Retention was good in all phases of the study. Specifically, 87.06% of the participants attended to the second session, 75.29% to the third session, and 64.71% to the last session. Following the v-Body Project protocol, all participants who were absent from the sessions attended a summary session [13,22]. The attrition varied from 34.12% to 45.88% at post-intervention and 6-month follow-ups, respectively.

The efficacy of the v-Body Project over all timepoints is shown in Table 2. Results demonstrated improvements in all outcomes at post-intervention. Large effect sizes were found for ED symptoms, body dissatisfaction, the thin ideal internalization, negative affect, and body appreciation, which were maintained up to 6-months. A small effect size was identified for self-esteem and the effect was maintained up to 6-months.

Regarding the main outcome (i.e., ED symptoms), we found significant reductions with large effects (F_(1.762, 148.035)_ = 32.864; *p* < 0.001; partial-*η*^2^ = 0.281). Bonferroni post-hoc analysis identified differences in ED symptom scores between the baseline and all timepoints, and no differences were identified between post-intervention and 1- and 6-month follow-up scores (Table 2). A similar pattern of results was obtained for body dissatisfaction. We found significant reductions with large effects on body dissatisfaction (F_(1.904, 159.964)_ = 68.671; *p* < 0.001; partial-*η*^2^ = 0.450). Bonferroni post-hoc analysis identified differences in body dissatisfaction scores between the baseline and all timepoints. No differences were identified between post-intervention and 1- and 6-month follow-up scores (Table 2).

Regarding the thin ideal internalization, we found significant reductions with a large effect (F_(2.177, 182.890)_ = 35.865; *p* < 0.001; partial-*η*^2^ = 0.299). Bonferroni post-hoc analysis identified differences in the thin ideal internalization scores between the baseline and all timepoints. Differences were also found between post-intervention and 1-month follow-up (a small effect size). No differences were identified between post-intervention and 6-months follow-up, and between 1- and 6-months follow-ups (Table 2). A similar pattern of results was obtained for negative affect. We found significant reductions with a large effect (F_(2.514, 211.199)_ = 29.419; *p* < 0.001; partial-*η*^2^ = 0.259). Bonferroni post-hoc analysis identified differences in negative affect scores between the baseline and all timepoints. Differences were also found between post-intervention and 1-month follow-up (a small effect size). No differences were identified between post-intervention and 6-months follow-up, and between 1- and 6-months follow-ups (Table 2).

Results from self-esteem and body appreciation showed efficacy of the v-Body Project. Concerning self-esteem, an increase with a small effect was observed (F_(2.030, 170.543)_ = 5.020; *p* = 0.007; partial-*η*^2^ = 0.056). Bonferroni post-hoc analysis identified differences in self-esteem scores between the baseline and post-intervention and 6-months follow-up (small effect sizes). Differences were not found between the baseline and 1-month follow-up. No differences were identified between post-intervention and 1- and 6-months follow-ups, and between 1- and 6-months follow-ups (Table 2). With respect to body appreciation, we observed an increase with a large effect (F_(1.853, 155.681)_ = 39.481; *p* < 0.001; partial-*η*^2^ = 0.320). Bonferroni post-hoc analysis identified differences in body appreciation scores between the baseline and all timepoints, and no differences were identified between post-intervention, and 1- and 6-months follow-up scores (Table 2).

## 4. Discussion

The current study evaluated the efficacy of the v-Body Project intervention among Brazilian women. Consistent with our first hypothesis, we found significant decreases in ED symptoms, body dissatisfaction, the thin ideal internalization, and negative affect. Our second hypothesis was also supported with a significant increases in self-esteem and body appreciation. These findings are consistent with previous research with the in-person Body Project, which has been shown to be effective in lowering ED risk and increasing protective factors among young women up to 6 months [17,18] and up to 1-, 2-, and 3-years of follow-up [45,46].

In a recent scoping review, authors pointed out the impact of COVID-19 pandemic, with an increase of new ED cases, suggesting an urgent need to investigate virtually delivered ED treatment and prevention programs [47]. In the review, most prevention programs provide some benefit to users, compared to those who do not use any program, and may also be important to consider for the post-pandemic era. In addition, studies that evaluate differences of synchronous and asynchronous interventions, found a significant effect of the moderator’s presence guiding group discussions on reducing weight and shape concerns [48] and that participants reported feeling more comfortable sharing issues related to body image and food in these spaces than with family and friends [49].

The current study extends the evidence that the synchronous adaptation of the Body Project has a positive impact in reducing ED risk factors in Brazilian women, with a range from a large effect (*d* = 0.74–0.98) (e.g., the thin ideal internalization, ED symptoms, negative affect) to a very large effect in body dissatisfaction (*d =* 1.31) between the baseline and post-intervention, which is similar to initial studies that have already tested the v-Body Project [22,24,25]. Considering protective factors, a very large effect was found for body appreciation (*d* = 1.04) between the baseline and post-intervention, similarly to other in-person interventions that found significant increases compared to control conditions [16,17,18]. The few studies that evaluated self-esteem [18,24] found similar effects to the present study. We found a significant increase, but with a small effect size. It is unclear why small effect sizes were observed in self-esteem over the follow-ups. One possible explanation is the moderate scores of self-esteem of the participants at the baseline. In fact, the effect size observed between the baseline and post-intervention was *d* = 0.40. Taken together, these findings suggest that the v-Body Project might increase protective factors in addition to reduce risk factors for EDs onset.

Nonetheless, this study has limitations. One of the limitations of the current study is the lack of a control group. Therefore, it is not possible to ensure that the results are due to the intervention and not to other factors. Future studies may evaluate the v-Body Project’s effects compared to a control group. A second limitation is the small sample size that could have limited the power of the statistical analysis carried out. Third, only self-report measures were used. Future studies might implement some strategies (e.g., ecological momentary assessment, behavioral tracking of the thin ideal endorsement) to reduce self-report bias. Fourth, the follow-up was relatively short (6 months), given that some trials with v-Body Project have followed participants for up to 2 years [22]. Fifth, we did not collect data that could demonstrate how representative this sample is of Brazilian young adults, like race/ethnicity, socioeconomic status, and urban/rural distribution. Sixth, an adaptation framework was not employed, such as the Ecological Validity Model or an expert panel review, during the cross-cultural adaptation of the protocol. Despite these limitations, this study presents relevant progress about ED risk and protective factors, especially in Brazil, where studies were developed only in person and in specific contexts (e.g., women who attend technical and undergraduate degrees in a single institution) [17,18]. Although the data may not generalize to Brazilian young women, the v-Body Project reached participants from all regions of the country (See Table 1). A strength of the program is the impact in a different socio-cultural background, confirming previous studies that suggested that an intervention created in one cultural context can be effective in different cultures [16,17,18,22,24]. This is possible given that this program is participant-driven and allows participants to argue against the beauty ideal promoted in their local cultures. For example, in Session 1, group facilitators prompt participants to reflect on societal messages regarding the thin ideal appearance for young women, allowing them to articulate this ideal within their own cultural background.

## 5. Conclusions

The results of this pilot study suggest that the v-Body Project (“Body Project Brazil”) is a promising tool for ED prevention among young adult Brazilian women, offering a more accessible option, with lower costs for participants and a scalable alternative to in-person programs, while maintaining significant positive effects on key ED-related outcomes. This aligns with the motivation for developing virtual versions to enhance scalability while preserving group dynamics.

While a previous unmoderated internet version of the Body Project (i.e., e-Body Project) showed weaker and less persistent effects compared to the in-person version, the current study, using a virtually implemented group format with trained facilitators, demonstrates sustained efficacy up to 6 months. In theory, the absence of a group context in the e-Body Project could account for its reduced effectiveness. The group context is believed to enhance peer accountability, a key component for inducing dissonance [50]. The v-Body Project is a promising ED intervention given that preserves the interactive elements between participants and facilitators and allows scalability [22]. Future research may compare the v-Body Project with the in-person Body Project and the e-Body Project in a Brazilian sample, to determine the relative effectiveness of each version of the program.

The partnership with a non-governmental organization for recruitment also highlights a potential strategy for wider dissemination. In terms of scalability, it is essential to consider partnerships with Brazil’s *Sistema Único de Saúde* (SUS). Training facilitators—such as nurses, healthcare professionals, and social workers—could enable the program’s implementation within the Brazil’s public health system, which serves as the primary health care. Further research with control groups is warranted to confirm these findings and to further explore the long-term impact of the v-Body Project in diverse populations and cultural contexts.

## Figures and Tables

**Table 1:** Demographic characteristics of Brazilian young women who took part in the v-Body Project.

Variables	Statistics	Range
	M (SD)	Min–Max
Age (years)	22.55 (2.07)	18–25
Body dissatisfaction	6.88 (0.47)	5–7
Brazilian regions	N (%)	
North	5 (5.88%)	-
North East	13 (15.29%)	-
South	16 (18.82%)	-
Southeast	47 (55.29%)	-
Midwest	4 (4.72%)	-
Self-reported anxiety symptoms		
Yes	43 (50.59%)	-
No	24 (28.24%)	-
Don’t know/Not reported	18 (21.17%)	-
Self-reported depression symptoms		
Yes	37 (43.53%)	-
No	30 (35.30%)	-
Don’t know/Not reported	18 (21.17%)	-

Note: *n* = 85. M = mean; SD = standard deviation; N = absolute frequency; % = relative frequency (percentage).

**Table 2:** Means and standard deviations for outcome variables at each timepoint.

	Timepoints				Effect Sizes (CI95%)		
Variables	T1	T2	T3	T4	Cohen’s *d* T1–T2	Cohen’s *d* T2–T3	Cohen’s *d* T3–T4
ED symptoms	18.51 (10.31) ^a^	12.23 (6.71) ^a^	12.48 (6.93) ^a^	12.25 (6.69) ^a^	0.80 (0.45–1.15)	−0.03 (−0.21–0.14)	0.03 (−0.14–0.20)
Body dissatisfaction	30.41 (8.69) ^a^	21.89 (6.03) ^a^	21.71 (5.36) ^a^	22.45 (5.42) ^a^	1.31 (0.85–1.76)	0.03 (−0.18–0.24)	−0.11 (−0.31–0.09)
The thin ideal internalization	70.04 (15.01) ^a^	60.59 (12.37) ^a,b^	57.65 (11.46) ^a,b^	59.22 (11.66) ^a^	0.74 (0.42–1.07)	0.23 (−0.02–0.48)	−0.12 (−0.31–0.06)
Negative affect	28.92 (8.91) ^a^	21.73 (7.42) ^a,b^	23.72 (6.08) ^a,b^	22.92 (6.72) ^a^	0.98 (0.59–1.37)	−0.27 (−0.55–0.01)	0.11 (−0.11–0.32)
Self-esteem	15.88 (6.31) ^c^	18.08 (5.48) ^c^	17.69 (4.73)	18.08 (5.25) ^c^	−0.40 (−0.81–0.01)	0.07 (−0.17–0.32)	−0.07 (−0.27–0.13)
Body appreciation	29.27 (9.44) ^a^	36.66 (6.32) ^a^	36.37 (5.90) ^a^	35.51 (6.29) ^a^	−1.04 (−1.45–0.63)	0.04 (−0.17–0.25)	0.12 (−0.05–0.29)

Note: *n* = 85; T1 = baseline; T2 = post-intervention; T3 = 1-month follow up; and T4 = 6-months follow-up. ^a^ Significant differences (*p* < 0.001) between the baseline and all timepoints. ^b^ Significant differences (*p* < 0.05) between post-intervention and 1-month follow-up. ^c^ Significant differences (*p* < 0.05) between the baseline and post-intervention and 6-months follow-up.

## Data Availability

Data are available upon reasonable request.

## References

[1] Kazdin A.E., Fitzsimmons-Craft E.E., Wilfley D.E. (2017). Addressing critical gaps in the treatment of eating disorders. Int. J. Eat. Disord..

[2] American Psychiatric Association [APA] (2022). Diagnostic and Statistical Manual of Mental Disorders.

[3] Allen K.L., Byrne S.M., Oddy W.H., Crosby R.D. (2013). DSM-IV-TR and DSM-5 eating disorders in adolescents: Prevalence, stability, and psychosocial correlates in a population-based sample of male and female adolescents. J. Abnorm. Psychol..

[4] Arcelus J., Mitchell A.J., Wales J., Nielsen S. (2011). Mortality rates in patients with anorexia nervosa and other eating disorders. A meta-analysis of 36 studies. Arch. Gen. Psychiatry.

[5] Trindade A.P., Appolinario J.C., Mattos P., Treasure J., Nazar B.P. (2018). Eating disorder symptoms in Brazilian university students: A systematic review and meta-analysis. Braz. J. Psychiatry.

[6] de Matos A.P., Rodrigues P.R.M., Fonseca L.B., Ferreira M.G., Muraro A.P. (2021). Prevalence of disordered eating behaviors and associated factors in Brazilian university students. Nutr. Health.

[7] Santana D.D., Barros E.G., da Costa R.S., da Veiga G.V. (2017). Temporal changes in the prevalence of disordered eating behaviors among adolescents living in the metropolitan area of Rio de Janeiro, Brazil. Psychiatry Res..

[8] Galmiche M., Déchelotte P., Lambert G., Tavolacci M.P. (2019). Prevalence of eating disorders over the 2000–2018 period: A systematic literature review. Am. J. Clin. Nutr..

[9] Swanson S.A., Crow S.J., Le Grange D., Swendsen J., Merikangas K.R. (2011). Prevalence and correlates of eating disorders in adolescents. Results from the national comorbidity survey replication adolescent supplement. Arch. Gen. Psychiatry.

[10] Fairburn C.G., Bailey-Straebler S., Basden S., Doll H.A., Jones R., Murphy R., O’Connor M.E., Cooper Z. (2015). A transdiagnostic comparison of enhanced cognitive behaviour therapy (CBT-E) and interpersonal psychotherapy in the treatment of eating disorders. Behav. Res. Ther..

[11] Monteleone A.M., Pellegrino F., Croatto G., Carfagno M., Hilbert A., Treasure J., Wade T., Bulik C., Zipfel S., Hay P. (2022). Treatment of eating disorders: A systematic meta-review of meta-analyses and network meta-analyses. Neurosci. Biobehav. Rev..

[12] Streatfeild J., Hickson J., Austin S.B., Hutcheson R., Kandel J.S., Lampert J.G., Myers E.M., Richmond T.K., Samnaliev M., Velasquez K. (2021). Social and economic cost of eating disorders in the United States: Evidence to inform policy action. Int. J. Eat. Disord..

[13] Stice E., Rohde P., Shaw H., Gau J.M. (2020). Clinician-led, peer-led, and internet delivered dissonance-based eating disorder prevention programs: Effectiveness of these delivery modalities through 4-year follow-up. J. Consult. Clin. Psychol..

[14] Stice E., Onipede Z.A., Marti C.N. (2021). A meta-analytic review of trials that tested whether eating disorder prevention programs prevent eating disorder onset. Clin. Psychol. Rev..

[15] Stice E., Marti C.N., Spoor S., Presnell K., Shaw H. (2008). Dissonance and healthy weight eating disorder prevention programs: Long-term effects from a randomized efficacy trial. J. Consult. Clin. Psychol..

[16] Amaral A.C.S., Stice E., Ferreira M.E.C. (2019). A controlled trial of a dissonance-based eating disorders prevention program with Brazilian girls. Psicol. Reflex. Crít..

[17] Hudson T.A., Amaral A.C.S., Stice E., Gau J., Ferreira M.E.C. (2021). Dissonance-based eating disorder prevention among Brazilian young women: A randomized efficacy trial of the Body Project. Body Image.

[18] Resende T.R.O., Almeida M., dos Santos Alvarenga M., Brown T.A., de Carvalho P.H.B. (2022). Dissonance-based eating disorder prevention improves intuitive eating: A randomized controlled trial for Brazilian women with body dissatisfaction. Eat. Weight Disord..

[19] Almeida M., Brown T.A., Campos P.F., Amaral A.C.S., de Carvalho P.H.B. (2021). Dissonance-based eating disorder prevention delivered in-person after an online training: A randomized controlled trial for Brazilian men with body dissatisfaction. Int. J. Eat. Disord..

[20] Almeida M., Brown T.A., Campos P.F., Resende T.R.O., de Carvalho P.H.B. (2024). A randomized controlled trial of a dissonance-based eating disorder prevention intervention for body-dissatisfied Brazilian men: Results from a 1-year follow-up. Braz. J. Psychiatry.

[21] Almeida M., Santos C.G., de Oliveira Júnior M.L., Resende T.R.O., Blashill A.J., Brown T.A., de Carvalho P.H.B. (2024). Dissonance-based eating disorder prevention for body-dissatisfied Brazilian cisgender gay and bisexual men: A randomized controlled trial with a 1-year follow-up. Int. J. Eat. Disord..

[22] Ghaderi A., Stice E., Andersson G., Enö Persson J., Allzén E. (2020). A randomized controlled trial of the effectiveness of virtually delivered Body Project (vBP) groups to prevent eating disorders. J. Consult. Clin. Psychol..

[23] Stice E., Rohde P., Durant S., Shaw H. (2012). A preliminary trial of a prototype Internet dissonance-based eating disorder prevention program for young women with body image concerns. J. Consult. Clin. Psychol..

[24] Ergut N., Keser E. (2022). Testing the efficacy of the Virtual Body Project in a sample of Turkish female university students using a randomized controlled trial. Klin. Psikol. Derg..

[25] Stice E., Bohon C., Shaw H., Desjardins C.D. (2023). Efficacy of virtual delivery of a dissonance-based eating disorder prevention program and evaluation of a donation model to support sustained implementation. J. Consult. Clin. Psychol..

[26] Wisting L., Stice E., Ghaderi A., Dahlgren C.L. (2023). Effectiveness of virtually delivered Body Project groups to prevent eating disorders in young women at risk: A protocol for a randomized controlled trial. J. Eat. Disord..

[27] Teixeira A.A., Roque M.A., de Freitas A.A., Dos Santos N.F., Garcia F.M., Khoury J.M., Garcia F.D. (2021). The Brazilian version of the SCOFF questionnaire to screen eating disorders in young adults: Cultural adaptation and validation study in a university population. Braz J Psychiatr.

[28] Nunes M.A., Camey S., Olinto M.T.A., Mari J.J. (2005). The validity and 4-year test-retest reliability of the Brazilian version of the Eating Attitudes Test-26. Brazilian. Braz. J. Med. Biol. Res..

[29] Garner D.M., Olmsted M.P., Bohr Y., Garfinkel P.E. (1982). The Eating Attitudes Test: Psychometric features and clinical correlates. Psychol. Med..

[30] Welch E., Lagerström M., Ghaderi A. (2012). Body shape questionnaire: Psychometric properties of the short version (BSQ-8C) and norms from the general Swedish population. Body Image.

[31] da Silva W.R., Dias J.C.R., Maroco J., Campos J.A.D.B. (2014). Confirmatory factor analysis of different versions of the Body Shape Questionnaire applied to Brazilian university students. Body Image.

[32] Thompson J.K., Van den Berg P., Roehrig M., Guarda A.S., Heinberg L.J. (2004). The sociocultural attitudes towards appearance Scale-3 (SATAQ-3): Development and validation. Int. J. Eat. Disord..

[33] Amaral A.C.S., Ribeiro M.S., Conti M.A., Ferreira C.S., Ferreira M.E.C. (2013). Psychometric evaluation of the sociocultural attitudes towards appearance Questionnaire-3 among Brazilian young adults. Span. J. Psychol..

[34] Watson D., Clark L.A. (1992). Affects separable and inseparable: On the hierarchical arrangement of the negative affects. J. Pers. Soc. Psychol..

[35] Amaral A.C.S. (2015). Imagem Corporal de Adolescentes: Descrição e Intervenção Preventiva em Âmbito Escolar. Ph.D. Thesis.

[36] Rosenberg M. (1965). Rosenberg Self-Esteem Scale (RSES) [Database Record].

[37] Hutz C.S., Zanon C. (2011). Revisão da adaptação, validação e normatização da escala de autoestima de Rosenberg. Interam. J. Psychol. Assess..

[38] Avalos L., Tylka T.L., Wood-Barcalow N. (2005). The Body Appreciation Scale: Development and psychometric evaluation. Body Image.

[39] Swami V., Campana A.N., Ferreira L., Barrett S., Harris A.S., Tavares M.C. (2011). The Acceptance of Cosmetic Surgery Scale: Initial examination of its factor structure and correlates among Brazilian adults. Body Image.

[40] Parent M.C. (2013). Handling item-level missing data: Simpler is just as good. Couns. Psychol..

[41] Singer J.D., Willett J.B. (2003). Applied Longitudinal Data Analysis: Modeling Change and Event Occurrence.

[42] JASP Team (2024). JASP, Version 0.19.1.

[43] Field A. (2018). Discovering Statistics Using SPSS.

[44] Cohen J. (2013). Statistical Power Analysis for the Behavioral Sciences.

[45] Stice E., Butryn M.L., Rohde P., Shaw H., Marti C.N. (2013). An effectiveness trial of a new enhanced dissonance eating disorder prevention program among female college students. Behav. Res. Ther..

[46] Stice E., Rohde P., Butryn M.L., Shaw H., Marti C.N. (2015). Effectiveness trial of a selective dissonance-based eating disorder prevention program with female college students: Effects at 2-and 3-year follow-up. Behav. Res. Ther..

[47] Pellegrini D., Grennan L., Bhatnagar N., McVey G., Couturier J. (2022). Virtual prevention of eating disorders in children, adolescents, and emerging adults: A scoping review. J. Eat. Disord..

[48] Serdar K., Kelly N.R., Palmberg A.A., Lydecker J.A., Thornton L., Tully C.E., Mazzeo S.E. (2014). Comparing online and face-to-face dissonance-based eating disorder prevention. Eat. Disord..

[49] Kass A.E., Trockel M., Safer D.L., Sinton M.M., Cunning D., Rizk M.T., Taylor C.B. (2014). Internet-based preventive intervention for reducing eating disorder risk: A randomized controlled trial comparing guided with unguided self-help. Behav. Res. Ther..

[50] Green M., Scott N., Diyankova I., Gasser C. (2005). Eating disorder prevention: An experimental comparison of high level dissonance, low level dissonance, and no-treatment control. Eat. Disord..

